# Supplementary material for: Adopting a model of antimicrobial stewardship program to anti-tubercular treatment stewardship: A single-centre experience from a private tertiary care hospital in South India
Source: PLoS One. 2024 Nov 5;19(11):e0310493. doi: 10.1371/journal.pone.0310493 (PMC11537384; doi:10.1371/journal.pone.0310493)
Supplement: S1 File — (PDF) [file pone.0310493.s001.pdf]

ATT Stewardship Data Collection Form

Name of the patient: Age:

MRD No: Sex:

Date of Admission: Height:

IP/OP: Weight at start of ATT:

Location: History of weight loss:

Isolated/ non isolated: History of weight gain:

Department: Current weight:

Consultant:

ATT start date:

Date of Review:

Reason for admission:

Comorbidities

CLD

CKD

HIV

Others(specify):

Family history of TB:

Symptoms

Cough (>2 weeks)

Fever (>2 weeks)

Night sweats

Hemoptysis

Diagnostic tests performed: { Text box with information}

X Ray:

Histopathology:

HIV test:

IGRA:

Mantoux Test:

FL- LPA:

SL-LPA:

LC DST:

Specimen:

**OTHER TESTS**	DATE OF SENDING	DATE OF REPORTING	RESULT
SMEAR AFB/TB			
CULTURE AFB/TB			
GENE XPERT			
CULTURE AND SENSITIVITY TESTS			

Diagnosis (Probable/Definite):

Classification of Patient {Text box with information}

a) New case
b) Previously treated case

Classification of TB {Text box with information}

1.Pulmonary TB

2. Extra pulmonary TB:

a. TB Meningitis
b. Lymph Node TB
c. Pleural TB
d. Intestinal TB
e. Joint and bone TB
f. GU TB
g. CNS TB
h. ENT TB
i. Abdominal TB
j. Cutaneous TB
k. Latent TB

Vitals

RR:

PR:

BP:

Temp:

Lab Parameters

ESR: Body Fluid Cell Count:

SGOT: DC:

SGPT: Body Fluid ADA:

S.Urea: Body Fluid LDH:

S.Cr : Body Fluid Protein:

Uric Acid:

Drug Regimen {Text box with information of treatment}

Intensive phase ☐ Continuation phase ☐

Current Medications other than ATT Drugs:

Drug	Dose	Route	Frequency	Date of initiation	Duration	

ATT Drugs {Text box with information of dose and duration}

Drug	Dose	Route	Frequency	Date of initiation	Duration	Weight based dosing

Recommendation:

Compliance:

Comments:
