# Supplementary material for: Adopting a model of antimicrobial stewardship program to anti-tubercular treatment stewardship: A single-centre experience from a private tertiary care hospital in South India
Source: PLoS One. 2024 Nov 5;19(11):e0310493. doi: 10.1371/journal.pone.0310493 (PMC11537384; doi:10.1371/journal.pone.0310493)
Supplement: S2 File — (PDF) [file pone.0310493.s002.pdf]

Amrita Institute of Medical Sciences and Research Centre

(an ISO 9001/14001/18001/NABH/NABL/NAAC certified hospital)

ANTITUBERCULAR DRUG STEWARDSHIP COMMITTEE

A part of Antibiotic Stewardship Committee

RECOMMENDATION FORM

Dear Doctor:

The following information is presented to you for your review and evaluation regarding this patient's antitubercular therapy. These recommendations are based primarily on laboratory data and your clinical judgment of the patient's condition should be used to determine the best treatment course for the patient.

Allergies:_____________ Patient's Name:_________________________ MRD No: _______________Room:_______

Diagnostic modalities suggestive

Date	Site	Genexpert	Culture	Histopath	Radiology	Others

Current Anti TB Regimen

Date	Weight	ATT Drug	Days of Therapy

Clinical and Laboratory Monitoring

Date	Weight	LFT	RFT	Ophthalmology status	Others

Please consider the following change(s) to the antibiotic regimen:

1.

2.

3.

4.

5.

Antituberculosis Stewardship team:

Dr. Beena K V, Dr.Dipu T S, Dr.Merlin Moni, Dr.Akhilesh K, Dr.Subhash Chandra, Dr.Swathy S Samban, Dr.Abhinand, Dr.Razak, Ms.Chithira

NOTE: THIS IS NOT A PART OF THE PATIENT'S PERMANENT MEDICAL RECORD
