# Supplementary material for: Adopting a model of antimicrobial stewardship program to anti-tubercular treatment stewardship: A single-centre experience from a private tertiary care hospital in South India
Source: PLoS One. 2024 Nov 5;19(11):e0310493. doi: 10.1371/journal.pone.0310493 (PMC11537384; doi:10.1371/journal.pone.0310493)
Supplement: S1 Table — (DOCX) [file pone.0310493.s003.docx]

| Adverse Drug Reactions (ADRs) | n (%) |
| --- | --- |
| Drug induced hepatitis | 15 (8.72) |
| Vomiting | 1 (0.58) |
| Hyperuricemia secondary to pyrazinamide | 1 (0.58) |
| Joint pain | 1 (0.58) |
| Thrombocytopenia | 1 (0.58) |
| Neurotoxicity | 1 (0.58) |
