# Supplementary material for: Adopting a model of antimicrobial stewardship program to anti-tubercular treatment stewardship: A single-centre experience from a private tertiary care hospital in South India
Source: PLoS One. 2024 Nov 5;19(11):e0310493. doi: 10.1371/journal.pone.0310493 (PMC11537384; doi:10.1371/journal.pone.0310493)
Supplement: S2 Table — (DOCX) [file pone.0310493.s004.docx]

| **Characteristic** | **Dose decrease** N = 12*^a^* | **No adjustment** N = 145*^a^* | **Dose increase** N = 15*^a^* | **p-value***^b^* |
| --- | --- | --- | --- | --- |
| Age |  |  |  | 0.02 |
| Children (0 – 17 years) | 0 (0%) | 9 (90%) | 1 (10%) |  |
| Young adults (18 – 39 years) | 0 (0%) | 30 (97%) | 1 (3.2%) |  |
| Middle age (40 – 59 years) | 5 (7.6%) | 56 (85%) | 5 (7.6%) |  |
| Old age (60 – 90 years) | 7 (11%) | 50 (77%) | 8 (12%) |  |
| *^a^*n (%) | | | | |
| *^b^*Chi square test for trend in proportion | | | | |
