# Supplementary material for: Adopting a model of antimicrobial stewardship program to anti-tubercular treatment stewardship: A single-centre experience from a private tertiary care hospital in South India
Source: PLoS One. 2024 Nov 5;19(11):e0310493. doi: 10.1371/journal.pone.0310493 (PMC11537384; doi:10.1371/journal.pone.0310493)
Supplement: S3 Table — (DOCX) [file pone.0310493.s005.docx]

| S.NO | AGE | SEX | WEIGHT(kg) | COMORBIDITIES | DRUGS | INAPPROPRIATENESS | RECOMMENDATION |
| --- | --- | --- | --- | --- | --- | --- | --- |
| 1 | 22 | M | 69.5 | HTN, CKD-5 | HRE | FREQUENCY | KINDLY CONSIDER OPTIMIZING THE FREQUENCY OF ETHAMBUTOL 1000MG OD TO 1000MG THRICE WEEKLY ACCORDING TO CREATININE CLEARANCE |
| 2 | 50 | M | 50 | CKD-4, IDA | HRZE | FREQUENCY | KINDLY CONSIDER OPTIMIZING THE FREQUENCY OF PYRAZINAMIDE 1000MG OD TO 1000MG THRICE WEEKLY ACCORDING TO CREATININE CLEARANCE |
| 3 | 76 | M | 63 | DM, HTN, DLP, CKD-4 | HRZE | DOSE | CONSIDER OPTIMIZING THE DOSE OF PYRAZINAMIDE AND ETHAMBUTOL BASED ON PATIENT BODY WEIGHT. PYRAZINAMIDE 750MG TO 1500MG, ETHAMBUTOL 600MG TO 1200MG |
| 4 | 42 | F | 65 | NIL | HRZE | DOSE | KINDLY CONSIDER OPTIMIZING THE DOSE OF ATT DRUGS ACCORDING TO PATIENTS BODY WEIGHT. R 450MG TO 600MG, Z 750MG TO 1500MG, E 800MG TO 1200MG |
| 5 | 41 | M | 39 | NIL | HRZE | DRUG | KINDLY CONSIDER MODIFIED ATT REGIMEN AS RIFAMPICIN SHOWS INTERMEDIATE RESISTANCE |
| 6 | 64 | M | 75 | TYPE 2 DM | HRZE | DOSE | KINDLY CONSIDER OPTIMIZING THE DOSE OF ISONIAZID 375MG TO 300MG AND RIFAMPICIN 750MG TO 600MG |
| 7 | 80 | M | 57.4 | DM,HTN | HRZE | DOSE | CONSIDER OPTIMIZING THE DOSE OF ATT ACCORDING TO PATIENT WEIGHT. Z 750MG TO 1500MG AND E 800MG TO 1200MG |
| 8 | 20 | M | 52 | NIL | HRZE | INDICATION | KINDLY CONSIDER SENDING REPEAT SPUTUM GENEXPERT |
| 9 | 69 | M | 59 | CA TONGUE | HRE | DRUG | KINDLY CONSIDER ADDING PYRAZINAMIDE |
| 10 | 79 | F | 44 | DM,HASHIMOTO ENCEPHALOPATHY | HRZE | DOSE | CONSIDER MODIFYING THE DOSE OF ETHAMBUTOL 1000MG TO 800 MG |
| 11 | 58 | M | 58 | CLD CHILD A | HRE, LEVOFLOXACIN | DOSE | CONSIDER OPTIMIZING THE DOSE OF ETHAMBUTOL 1500MG TO 1200MG |
| 12 | 48 | M | 87 | NIL | HRE, LEVOFLOXACIN | DOSE | AS PER NTEP FOR WEIGHT BAND>75KG , DOSE OF ETHAMBUTOL CAN BE INCREASED FROM 1000MG TO1600 MG AND DOSE OF LEVOFLOXACIN CAN BE INCREASED FROM 500MG TO 750MG IF ADEQUATE DOSE IS NOT AVAILABLE CONSIDER CHANGING TO FDC REGIMEN, ADDING PYRIDOXINE CAN ALSO BE CONSIDERED SINCE THE PATIENT IS ON ISONIAZID |
| 13 | 54 | M | 76 | NIL | HRZE | DOSE | KINDLY CONSIDER OPTIMISING THE DOSE OF ISONIAZID FROM 450MG TO 300MG |
| 14 | 78 | M | 60 | HTN,DM-2, PARKINSON,  THROMBOCYTOPENIA,  SPLENOMEGALY,  DIABETIC NEPHROPATHY | HRZE | DOSE | OPTIMISE ETHAMBUTOL DOSE FROM 1000MG TO 1200MG BASED ON WEIGHT. |
| 15 | 53 | F | 50 | DM-2,HTN,CKD,CAD,DLP | H,R,Z,E | INDICATION | KINDLY CONSIDER SENDING THE DIAGNOSTIC WORKUP FOR RULING OUT GENITOURINARY TB |
| 16 | 72 | M | 69.3 | DM-2,HTN,CAD,COPD | H,R,STREPTOMYCIN,  E,OFLOXACIN | DRUG | KINDLY CONSIDER DISCONTINUING STREPTOMYCIN IF RIFANPICIN GETS TOLERATED |
| 17 | 58 | M | 60 | TRAUMATIC PARAPLEGIA | INH,R,E,Z,PYRIDOIME | DOSE | KINDLY CONSIDER THE ATT TREATMENT BASED ON THR PATIENT'S BODY WEIGHT. H 450 MG TO 300MG |
| 18 | 39 | M | 55 | TB MENINGITIS AND DISSEMINATD  MILIARY TB, RHEUMATOID  ARTHRITIS | HRE | DOSE | OPTIMISE RIFAMPICIN DOSE FROM 450MG TO 600MG BASED ON WEIGHT. |
| 19 | 48 | M | 59 | DM-2,DLP,CAD,TMT POSITIVE | HRE | DRUG | MEDICINE PRESCRIBED FOR ONE YEAR WITHOUT Z DUE TO SGPT ELEVATED. TO REPEAT LFT, IF Z IS NOT TOLERATED CONSIDER ADDING LEVOFLOX |
| 20 | 40 | M | 80 | NIL | HRE | DOSE | OPTIMISE ETHAMBUTOL DOSE FROM 600MG TO 1200MG BASED ON WEIGHT. |
| 21 | 56 | F | 80 | HTN,DIABETES,HYPOTHYROIDISM | HRZE | DOSE | OPTIMISE ETHAMBUTOL DOSE FROM 1000MG TO 1600MG AND PYRAZINAMIDE DOSE FROM 1000MG TO 2000MG BASED ON WEIGHT. |
| 22 | 53 | M | 43.7 | NIL | HRZE | OTHER | IF CULTURE AFB/TB IS POSITIVE, KINDLY CONSIDER DOING FIRST LINE ATT DRUG SUSCEPTIBILITY TEST AS MONO DRUG RESISTANCE ARE INCREASING |
| 23 | 68 | M | 66 | UNCONTROLLED TYPE 2 DIABETES  MELLITUS SINCE 23 YEARS -DIABETIC RETINOPATHY + NEPHROPATHY(CHRONIC  KIDNEY DISEASE) SINCE PAST 4 YEARS 2.SYSTEMIC HYPERTENSION SINCE 13 YEARS | E,Levoflox | DOSE | KINDLY CONSIDER DOSE ADJUSTMENT OF LEVOFLOX ACCORDING TO CREATININE CLEARANCE [CRCL 21ML/MIN(CR: 3.21, WT 66KG)] LEVOFLOX 500MG ONCE IN 48 HOURS TO 750MG ONCE IN 48 HOURS |
| 24 | 25 | F | 39 | NIL | E,Levoflox | DRUG | AS SGOT SGPT IS NORMAL, AND NO COMORBIDITIES, CAN RECHALLENGE WITH HRZE AND ONLY ONE LAB REPORT SHOWS ELEVATION |
| 25 | 89 | F | 40 | HTN, TDM, DLP | HRZE | DOSE | KINDLY OPTIMIZE THE DOSE OF ETHAMBUTOL 1200MG TO 800MG |
| 26 | 58 | M | 60 | SEIZURE, DLP | STREP AND ETHAMBUTOL | DRUG | IN VIEW OF DISSEMINATED TB, KINDLY CONSIDER ADDING LINEZOLID 600MG BD TO THE CURRENT MODIFIED TB TREATMENT UNTIL THE ATT INDUCED HEPATITIS RESOLVES. |
| 27 | 73 | M | 66 | NIL | HRZE | DRUG,DOSE | OPTIMISE DOSE OF ISONIAZID FROM 150MG TO 300MG AND DOSE OF RIFAMPICIN FROM 450MG TO 600MG BASED ON WEIGHT. KINDLY CONSIDER CHANGING TO MODIFIED ATT REGIMEN IN V/O DERANGED RFT. STOP ETHAMBUTOL AND PYRAZINAMIDE |
| 28 | 70 | M | 80 | HTN, CKD, CAD, DYSLIPIDEMIA | HREZ+levofloxacin | DRUG,DOSE | KINDLY CONSIDER STOPPING LEVOFLOX. KINDLY CONSIDER OPTIMISING THE DOSE OF E 800MG TO 1600MG |
| 29 | 17 | F | 38.6 | NIL | HRZE | DOSE | THE DOSE OF RIFAMPICIN WAS INAPPROPRIATE. RIFAMPICIN 450MG TO 600MG |
| 30 | 81 | M | 60 | CAD, ASTHMA, COPD | HRZE | DOSE | THE DOSE OF RIFAMPICIN WAS INAPPROPRIATE. R 450MG TO 600MG |
| 31 | 59 | M | 62 | CCHD/EISENMENGER, CAD, DYSLIPIDEMIA | HRZE | DOSE | THE RIFAMPICIN DOSE NEED TO CORRECTED. R 300MG TO 600MG |
| 32 | 67 | M | 61 | CAD, HTN, TYPE II DM | HE, LEVO | DOSE | KINDLY CONSIDER OPTIMISING DOSE OF ETHAMBUTOL. ETHAMBUTOL 1500MG TO 1200MG |
| 33 | 36 | M | 64 | CLD WITH PORTAL HTN, CHRONIC ANEMIA | R,E+LEVOFLOX, STREPTOMYCIN | DRUG | KINDLY CONSIDER STOPPING RIFAMPICIN IN VIEW WORSENING LFT (THREE FOLD INCREASE) |
| 34 | 57 | M | 57 | HTN, POST RENAL TRANSPLANT | HEZ | DRUG | KINDLY CONSIDER MODIFIED ATT REGIMEN DERANGED LFT AND RFT |
| 35 | 73 | F | 60 | HTN | HRZE | DOSE | KINDLY OPTIMIZE THE DOSE OF ETHAMBUTOL 1500MG TO 1200MG AND PYRAZINAMIDE 2000MG TO 1500MG |
| 36 | 71 | M | 57 | TYPE 2 DM ,SHTN, CKD AND OLDTUBERCULOSIS | HRZE | DRUG, DOSE,  FREQUENCY | KINDLY CONSIDER MODIFIED ATT REGIMEN : HRLE. KINDLY CONSIDER OPTIMISING THE DOSE OF LEVOFLOX 750 MG OD TO 750MG ON EVERY ALTERNATE DAYS. KINDLY CONSIDER OPTIMISING THE DOSE OF ETHAMBUTOL 600MG TO 800MG |
